# The weight of office? A systematic scoping review of mental health issues and risk factors in elected politicians across democratic societies

**DOI:** 10.1192/j.eurpsy.2025.537

**Published:** 2025-08-26

**Authors:** A. Smith, S. Hachen, A. Weinberg, P. Falkai, S. Guttormsen, M. Liebrenz

**Affiliations:** 1 Department of Forensic Psychiatry; 2 University of Bern, Bern; 3 School of Health and Society, University of Salford, Salford, Switzerland; 4 LMU Clinic, Ludwig Maximilian University of Munich Public university in Munich, Germany, Munich, Germany; 5 Institute of Medical Education, University of Bern, Bern, Switzerland

## Abstract

**Introduction:**

The mental health status and capacity to govern of democratically-elected politicians have become significant topics of interest, which have attracted speculation in the media and beyond. In fulfilling demanding and high-stress positions, politicians could encounter distinctive risk factors that may harm their mental wellbeing, yet existing research literature about this topic remains underexplored.

**Objectives:**

This scoping review aimed to systematically examine the breadth of available evidence on mental health issues and risk factors affecting democratically-elected politicians and to identify future research needs.

**Methods:**

Using pre-defined eligibility criteria based on JBI guidelines, a systematic keyword search was conducted in May 2024 of MEDLINE, Scopus, and APA PsycNet, supplemented by snowballing techniques. Only studies reporting primary, empirical evidence on mental ill-health or risk factors with adverse psychological correlates from serving politicians in “Full” or “Flawed” democracies (per the Democracy Index) were included from 1999-2024. Titles and abstracts were screened and the full-text of potentially eligible literature was assessed before data extraction and synthesis.

**Results:**

Eighteen sources met the eligibility criteria, cumulatively encompassing ~3,500 politicians across seven democracies, namely: Australia, Canada, the Netherlands, Norway, New Zealand, the United Kingdom, and the United States. Four sources (22.2%) explored general psychopathology trends, revealing varying but sizeable rates of mental ill-health and high-risk alcohol consumption. The other fourteen studies (77.8%) provided evidence on risk factors; twelve underlined the psychological toll of violence and two investigations highlighted the injurious effects of specific occupational conditions. Notably, exposure to violence often precipitated detrimental mental health outcomes, with certain data indicating a disproportionate impact on female officeholders.

**Conclusions:**

Existing research literature suggests that democratically-elected politicians face considerable mental health challenges, especially from the effects of violence. However, there are notable research gaps with a paucity of reliable prevalence estimates, intervention studies, and work on national leaders. Equally, the underrepresentation of numerous democratic countries accentuates the need for a more diverse evidence-base to better support the mental wellbeing of politicians worldwide.

**Disclosure of Interest:**

None Declared

